# High resolution DOA estimation of acoustic plane waves: An innovative comparison among Cuckoo search heuristics and subspace based algorithms

**DOI:** 10.1371/journal.pone.0268786

**Published:** 2022-06-16

**Authors:** Nauman Ahmed, Huigang Wang, Rizwan Ahmad, Ali Ahmed Salem, Rahisham Abd Rahman, Muhammad Kashif, Shahzad Arshad, Kwan. Y. Lau

**Affiliations:** 1 School of Marine Science and Technology, Northwestern Polytechnical University XiAn, Shaanxi, China; 2 Faculty of Electrical and Electronic Engineering, University Tun Hussein Onn Malaysia, Batu Pahat, Malaysia; 3 Institute of High Voltage and High Current, School of Electrical Engineering, Universiti Teknologi Malaysia, Johor Bahru, Malaysia; 4 Department of Electrical Engineering, Sana’a University, Sana’a, Yemen; 5 Department of Electrical and Computer Engineering, COMSATS University Islamabad, Attock Campus, Attock, Pakistan; 6 Aerospace and Aviation (A&AC) Campus Kamra, Air University, Islamabad, Pakistan; Torrens University Australia, AUSTRALIA

## Abstract

SONAR signal processing plays an indispensable role when it comes to parameter estimation of Direction of Arrival (DOA) of acoustic plane waves for closely spaced target exclusively under severe noisy environments. Resolution performance of classical MUSIC and ESPRIT algorithms and other subspace-based algorithms decreases under scenarios like low SNR, smaller number of snapshots and closely spaced targets. In this study, optimization strength of swarm intelligence of Cuckoo Search Algorithm (CSA) is accomplished for viable DOA estimation in different scenarios of underwater environment using a Uniform Linear Array (ULA). Higher resolution for closely spaced targets is achieved using smaller number of snapshots viably with CSA by investigating global minima of the highly nonlinear cost function of ULA. Performance analysis of CSA for different number of targets employing estimation accuracy, higher resolution, variance analysis, frequency distribution of RMSE over the monte Carlo runs and robustness against noise in the presence of additive-white Gaussian measurement noise is achieved. Comparative studies of CSA with Root MUSIC and ESPRIT along with Crammer Rao Bound analysis witnesses better results for estimating DOA parameters which are further endorsed from the results of Monte Carlo simulations.

## 1. Introduction

Direction of arrival has remained an active topic for researchers in array signal processing for its vast applications in the field of electromagnetic signal processing, seismic exploration, acoustic signal processing, speech signal processing, defense (warfare), and biomedical imaging [1–4]. Acoustics plays a pivotal role in underwater wireless communication due to its nature of robust propagation in water as compared to electromagnetic waves.

DOA estimation for the pool of practical problems restraints the application of conventional subspace based algorithms due to limited number of snapshots and low SNR for closely spaced targets. CBF and spectral search algorithms are reliable for the applications involving greater number of snapshots, higher SNR and incoherent sources hence, it is challenging to apply these algorithms for DOA estimation. Maximum Likelihood (ML) [5] is another approach for parameter estimation of DOA having higher computational complexity which restrains its implementation for such problems [6–9]. Estimation accuracy in DOA is highly dependent on signal power and rate of transmission so, low SNR based DOA estimation is an innovative and challenging task in real time applications [10–13].

Recently, a pool of robust optimization algorithms have been introduced for DOA estimation such as Genetic Algorithm (GA) [14], Differential Evolution (DE) method [15], Particle Swarm Optimization (PSO) [16], Seeker Optimization Algorithm (SOA) [17], Sine Cosine algorithm (SCA) [18], Invasive Weed Optimization (IWO) [19], Squirrel Search Algorithm (SSA) [20], etc. In [14], GA is analyzed showing better results for the DOA estimation against its counterparts i.e. ML and MUSIC algorithm in terms of robustness against noise, computational cost and number of snapshots. A modified version of GA applied to highly nonlinear function estimating DOA parameters is presented in [21]. In [16], the PSO algorithm develops the mechanism to estimate the parameters of a multimodal function. In [22], PSO ML estimator shows very healthy and reliable results as compared to conventional parameter estimation techniques for DOA. Using Ant Colony Optimization (ACO) by extending the pheromone, DOA parameters are estimated in [23] with very good results and low computational complexity. In [24], Artificial Bee Colony (ABC) algorithm is used to achieve higher statistical performance. In 2019, Squirrel Search Algorithm (SSA) was proposed which is a novel numerical optimization algorithm. It focuses on foraging and gliding behavior of flying squirrels to determine their efficient way of locomotion. Gliding is a powerful technique used by small mammals for traveling long distances. Present work mathematically models this behavior to comprehend the process of optimization. These features may be helpful to improve convergence, reduce the number of iterations of the SSA algorithm and in determining ML DOA estimation [20].

All the above Evolutionary Algorithms (EA) perform well in locating single-dimensional optimization problem but fail to provide multiple solutions. Several approaches have been exploited in evolutionary algorithms to achieve multimodal optimization, such as fitness sharing [25, 26], deterministic crowding [27], probabilistic crowding [26, 28], clustering based niching [29], clearing procedure [30], species conserving genetic algorithm [31] and elitist-population strategies [32]. However, problems may still exist while implementing these strategies such as tuning niching parameters, retaining perceived solution in a run, extra computational costs and poor scalability for multidimensional problems. Moreover, these methods are hatched for spanning the search capacities of popular evolutionary algorithms such as GA and PSO, which fail to exploit the balance between exploration and exploitation, exclusively for multimodal objective functions [33]. Furthermore, they do not probe whole search span productively and most of the time do not converge. Recently, a novel nature-inspired algorithm known as Cuckoo Search Algorithm (CSA) [34], has been introduced for multidimensional optimization problems. The CSA is based on constrained brood-parasitic strategy of some cuckoo birds. One of the most significant characteristic of CSA is the employment of Levy flights to construct new solutions. Using this approach, the solutions are modified by adopting many small steps resulting in improvement of the relationship between exploration and exploitation [35]. Recent studies revealed that the CSA is far more efficient than PSO and GA [36]. Such characteristics have prompted the use of CSA to manipulate many engineering problems such as mesh generation [37], embedded systems [38], steel frame design [39], scheduling problems [40], thermodynamics [41] and distribution networks [42].

In this study, the optimization strength of swarm intelligent approach of the Cuckoo Search Algorithm (CSA) is accomplished for viable DOA estimation in different scenarios of underwater environment using Uniform Linear Array (ULA). The high resolution for closely spaced targets is achieved using fewer snapshots viably with CSA by investigating the global minima of the highly non-linear cost function of ULA. Performance analysis is conducted for the different number of targets employing estimation accuracy, resolution ability, frequency distribution of RMSE over the Monte Carlo runs and robustness against noise in the presence of additive white Gaussian measurement noise reveals better results. Comparative studies with Root MUSIC and ESPRIT counterparts along with Crammer Rao Bound analysis witnesses the worth of the scheme for estimating DOA parameters which are further endorsed from Monte Carlo simulation results.

The rest of the paper is organized as: In section II the data model for uniform linear array (ULA) is defined. The theoretical principle of the CSA algorithm for DOA estimation is described in section III. Performance analysis of algorithms in terms of RMSE is illustrated in section IV. Potential use and limitations are discussed in section V. The final section explains the main contributions of the proposed study.

## 2. Data model

**Fig 1:**
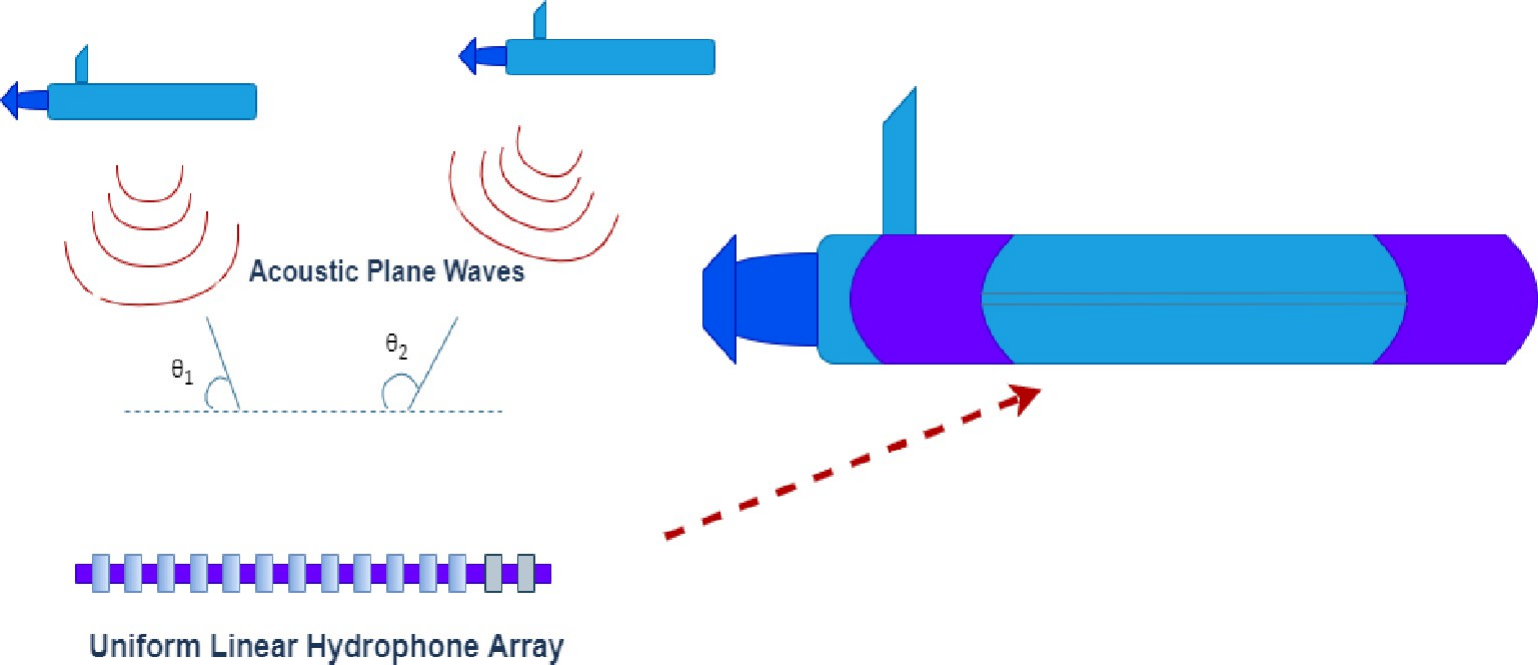
DOA estimation model.

In this work, the DOA model was estimated as shown in Fig 1. a ULA of hydrophones is used for DOA estimation of underwater multiple targets. A ULA having the characteristics of impinged plane waves from the far-field region is phase-shifted versions of neighbor hydrophones. DOA of multiple targets can be expressed as:

$$\theta =[\theta_{1},\theta_{2},\theta_{3},\ldots .,\theta_{D}]$$ (1)

where *θ*_*D*_ is associated angle to *D*^*th*^ acoustic source.

$$z(t)=[z_{1}(t),z_{2}(t),\ldots .,z_{W}(t)]$$ (2)

$$s(\theta_{i})=[1,e^{\left(-jkdsin(\theta_{i})\right)},e^{\left(-jk2dsin(\theta_{i})\right)},\ldots .,e^{-jk(W-1)dsin(\theta_{i})}{]}^{T}$$ (3)

$$S(\theta )=[s(\theta_{1}),s(\theta_{2}),\ldots .,s(\theta_{D})]$$ (4)

where, $k=\frac{2\pi }{\beta }$, while *β* is wavelength. **z**(**t**) is the hydrophone’s output vector with dimension *W*x1 and can be called as array response.


z(t)=S(θ)y(t)+n(t) (5)

The steering matrix *S* of dimension *W*x*D* comprises the time delay entities of signals for each hydrophone. Where **n**(**t**) is additive white Gaussian noise of zero mean with the dimension *W*x1. The co-variance matrix is defined as:

$$R=E[z(t)z^{H}(t)]$$ (6)

When a finite number of snapshots are available, the above equation can be written as:

$$R=\frac{1}{K}\sum_{n=1}^{K}‍z(n)z^{H}(n)$$ (7)

Where *E*[.] and [.]^*H*^ are ensemble average and Hermitian operators. So the correlation matrix can be written as:

$$R=SR_{s}S^{H}+\sigma^{2}I_{D}$$ (8)

Where **R**_*y*_ is correlation associated with signal and *σ*^2^**I**_*D*_ is the noise correlation matrix. The actual signal received on the array would be:

$${z_{\theta }}_{a}(t)=S(\theta_{a})y(t)+n(t)$$ (9)

Whereas, the approximated signal after getting optimum solutions would be:

$${z_{\theta }}_{e}(t)=S(\theta_{e})y(t)$$ (10)

So, objective function will become,

$$f({\theta_{\theta }}_{e})=|{z_{\theta }}_{e}(t)-{z_{\theta }}_{a}(t){|}^{2}$$ (11)

The general goal of DOA estimation is a continuous optimization that is used to find the *θ*_*best*_ which satisfies:

$$f(\theta_{best})=\underset{\theta \epsilon S}{min}f(\theta_{e})$$ (12)

where *SϵR*^*D*^ and *f*(*θ*) comprises the cost values of corresponding solution *θ*. Therefore, the actual goal of the optimizer is to compute the associated argument for the minimum cost function. So, population of *N* individuals will be used to solve the optimization problem having *T* iterations (trials). The set of D-Dimensional vectors (total *N* vectors) for *i* iterations can be denoted as:

$$\theta_{1}(i),\theta_{2}(i),\theta_{3}(i),\ldots ,\theta_{N}(i)$$ (13)

Since Cuckoo Search Algorithm [and Deb2009], can solve the multidimensional optimization problems so, the best solution at iteration *i* can be found as:

$$\theta_{best}(i)=arg\underset{n=1,\ldots ,N}{min}f(\theta_{n}(i))$$ (14)

## 3. Basic principle of CSA

There are two steps which are performed to get the optimum solution. Both of these steps belong to global and local searches. The global search is associated with levy flights and the local search is associated with random walks. Following are the implementation steps of the Cuckoo Search Algorithm:

1. A population of solutions is chosen randomly in the lower and upper bounds.
2. Fitness of each solution is computed and the best solution is selected among them (current best).
3. Each solution from the population is modified by the equation: (Global Optimization).

$$\theta_{i}(n+1)=\theta_{i}(n)+0.01.s.(\theta_{i}(n)-\theta_{best})$$ (15)

where *s* is generated by symmetric Levy Distribution as:

$$s=U.|V{|}^{-1/\lambda }$$

and *λ* = 1.5 for all optimization problems.Where, **u** and **v** are calculated from normal distributions (*U*~*N*(0, *σ*^2^) and *V*~*N*(0,1)) and *σ*^2^ can be computed as

$$\sigma^{2}={\left\{\frac{\Gamma (1+\lambda )}{\lambda \Gamma [(1+\lambda )/2]}.\frac{sin(\pi \lambda /2)}{2^{(\lambda -1)/2}}\right\}}^{1/\lambda }$$

After modification, both previous and modified solutions are compared and one of them based on minimum fitness value is selected. The fitness value would be calculated by inserting the modified solution *θ*_*i*_(*n*+1) as *θ*_*e*_:

$$f({\theta_{\theta }}_{e})=|{z_{\theta }}_{e}(t)-{z_{\theta }}_{a}(t){|}^{2}$$ (16)

This step is known as greedy selection or elitist selection.

4. Next step is associated with the local search. In this operation, solutions are probabilistically selected and modified with a new value. This operation is performed by generating a random number *ε* within a range of [0,1]. If the number is less than *p*_*a*_ then solution is modified by (below equation) otherwise, the solution remains unchanged (most of the practical problems *p*_*a*_ = 0.2)

$$\theta_{i}(n+1)=\theta_{i}(n)+\varepsilon (\theta_{j}(n)-\theta_{k}(n))$$ (17)

**Fig 2:**
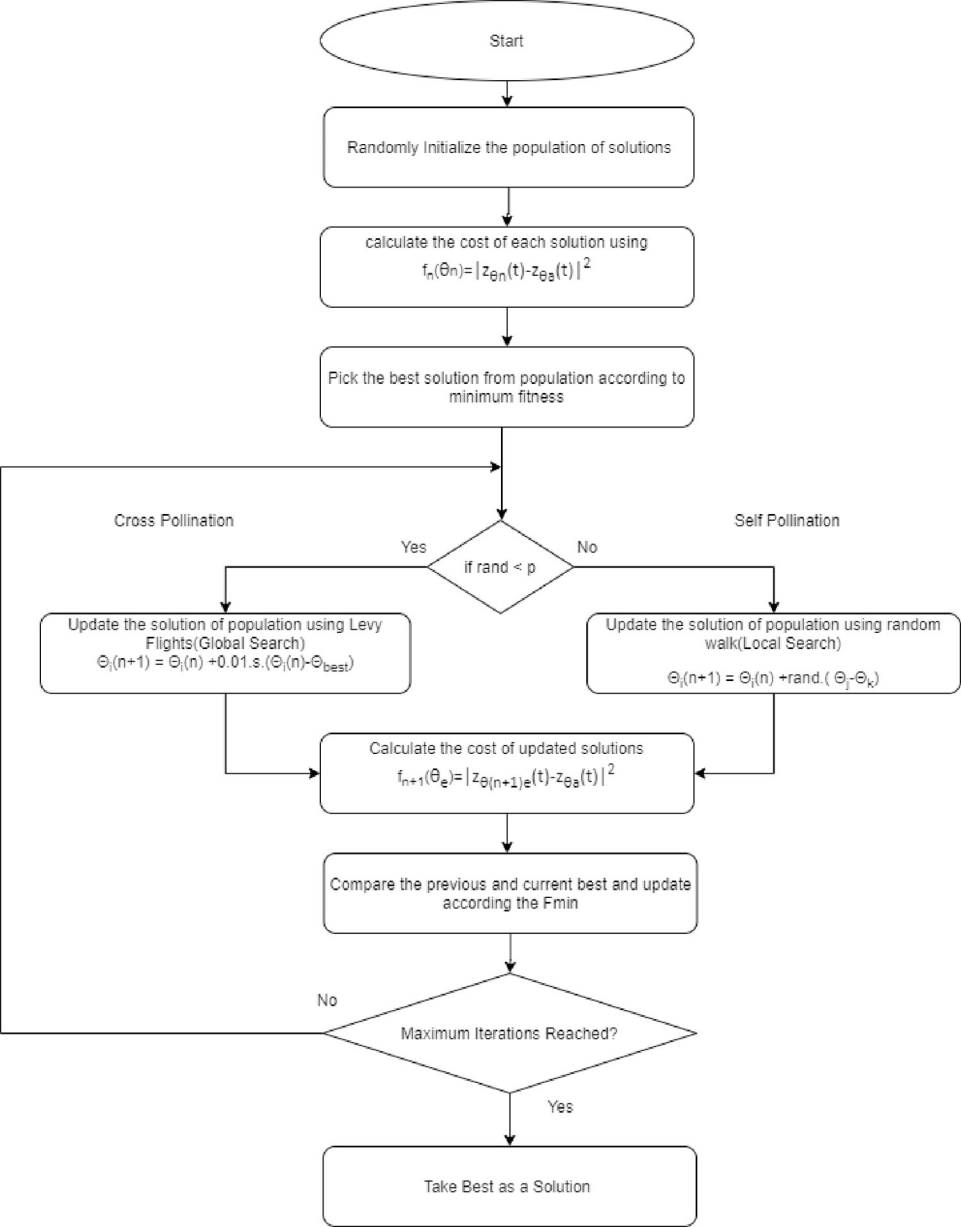
Flowchart of CSA.

Where *i* and *j* are chosen randomly from the population. After modification, an elitist selection strategy is performed once. The simulation conditions are listed in Table 1 and the CSA steps was illustrated in flowchart in Fig 2.

**Table 1:** Conditions for simulations.

Conditions for simulations	
Two Targets DOAs	30,35
Three Targets DOAs	30,40,50
Four Targets DOAs	10,20,30,40
Number of Snapshots	20
Number of MC Runs	300
Distance between hydrophones	*β*/2
Number of Hydrophones	8
Signal to Noise Ratio	5dB

## 4. Results and discussion

In this section, numerous simulations have been presented to assess the performance of CSA versus state of the art counterparts. The performance is evaluated in terms of estimation accuracy, convergence analysis, robustness against noise, and statistical analysis of RMSE over the Monte Carlo runs. The simulation conditions for different algorithms are given in Table 2. The performance metrics signifies the comprehensive analysis of algorithms as explained in the following sections:

**Table 2:** Estimation accuracy for two sources.

Schemes		CSA	ESPRIT	RMUSIC
*θ*_1_ = 30	Best	30.0024	30.1904	29.9188
	Average	29.9911	29.6800	26.1252
	Worst	29.5801	31.3427	87.6954
*θ*_1_ = 35	Best	34.9952	35.1052	34.7423
	Average	35.0037	35.6937	35.2464
	Worst	34.7150	75.6760	33.3052

### 4.1. Estimation accuracy

Estimation accuracy of Root MUSIC, ESPRIT and CSA algorithms are examined here by taking spatially separated targets and different levels of additive white Gaussian noise. The best, mean and worst numerical results have been presented for 300 independent Monte Carlo runs. It can be seen from Tables 2–4 that the CSA algorithm outperforms the state of the art schemes against all performance measures.

**Table 3:** Estimation accuracy for three sources.

Schemes		CSA	ESPRIT	RMUSIC
*θ*_1_ = 30	Best	29.9974	30.7139	29.9383
	Average	29.9896	29.0998	11.9609
	Worst	29.8207	60.3018	47.7647
*θ*_2_ = 40	Best	40.0057	40.0488	39.9289
	Average	40.0009	39.7917	38.3100
	Worst	39.8501	32.0663	31.806
*θ*_3_ = 50	Best	50.0055	49.9543	50.5934
	Average	49.9952	50.6858	48.3299
	Worst	49.2349	44.3527	31.8061

**Table 4:** Estimation accuracy for four sources.

Schemes		CSA	ESPRIT	RMUSIC
*θ*_1_ = 10	Best	10.0010	10.2908	10.0177
	Average	10.0025	6.7538	23.2058
	Worst	10.0512	85.3702	60.8888
*θ*_2_ = 20	Best	20.0052	19.6496	19.5832
	Average	20.0055	18.9887	14.2466
	Worst	20.1255	11.3950	34.1268
*θ*_3_ = 30	Best	29.9766	31.2964	9.9817
	Average	30.0030	30.6284	25.5408
	Worst	30.2080	23.8298	9.7894
*θ*_4_ = 40	Best	40.0017	40.1727	38.8653
	Average	39.9813	41.8516	37.2383
	Worst	39.4738	34.5331	36.6221

### 4.2. Convergence analysis

**Fig 3:**
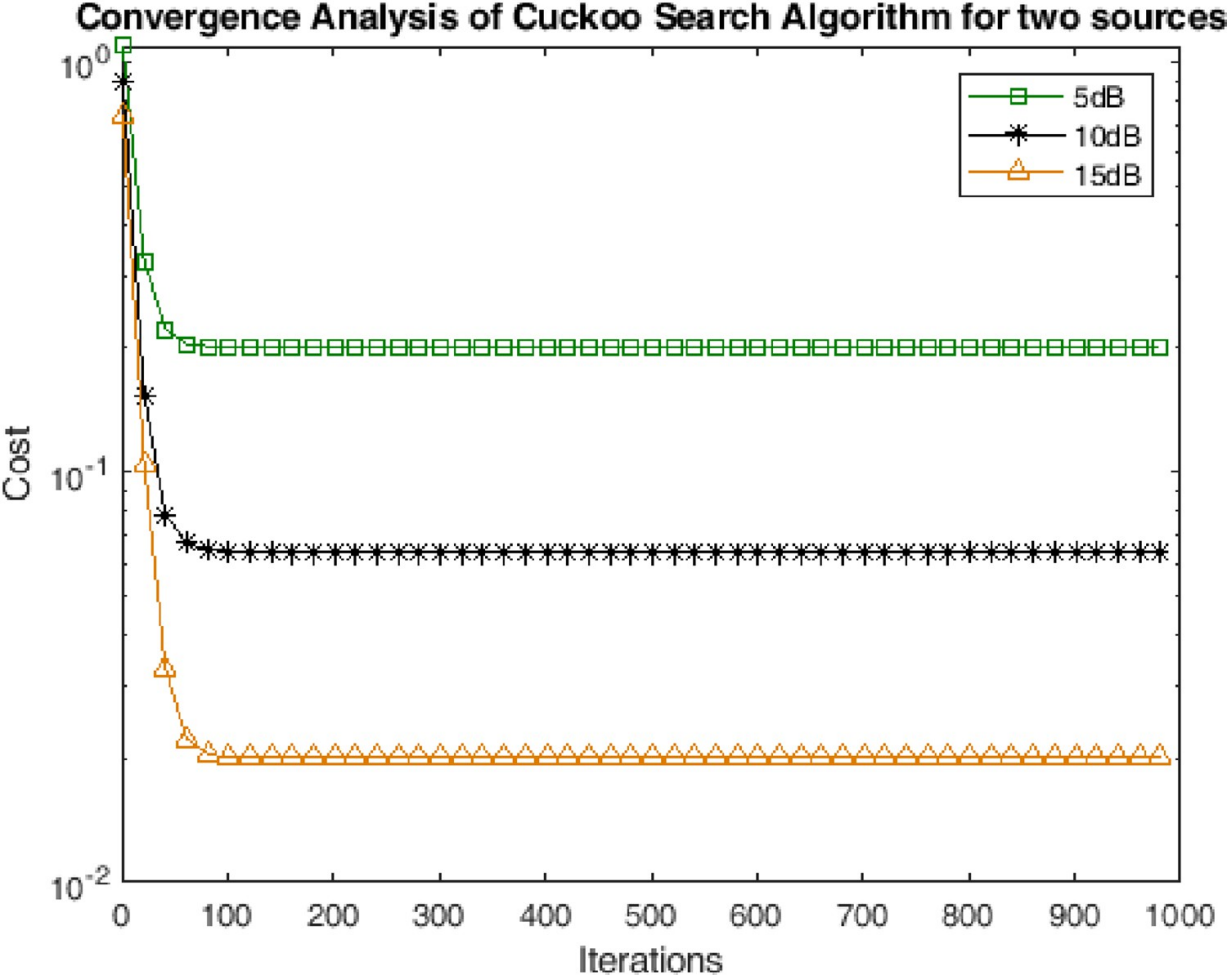
Convergence analysis for two sources.

**Fig 4:**
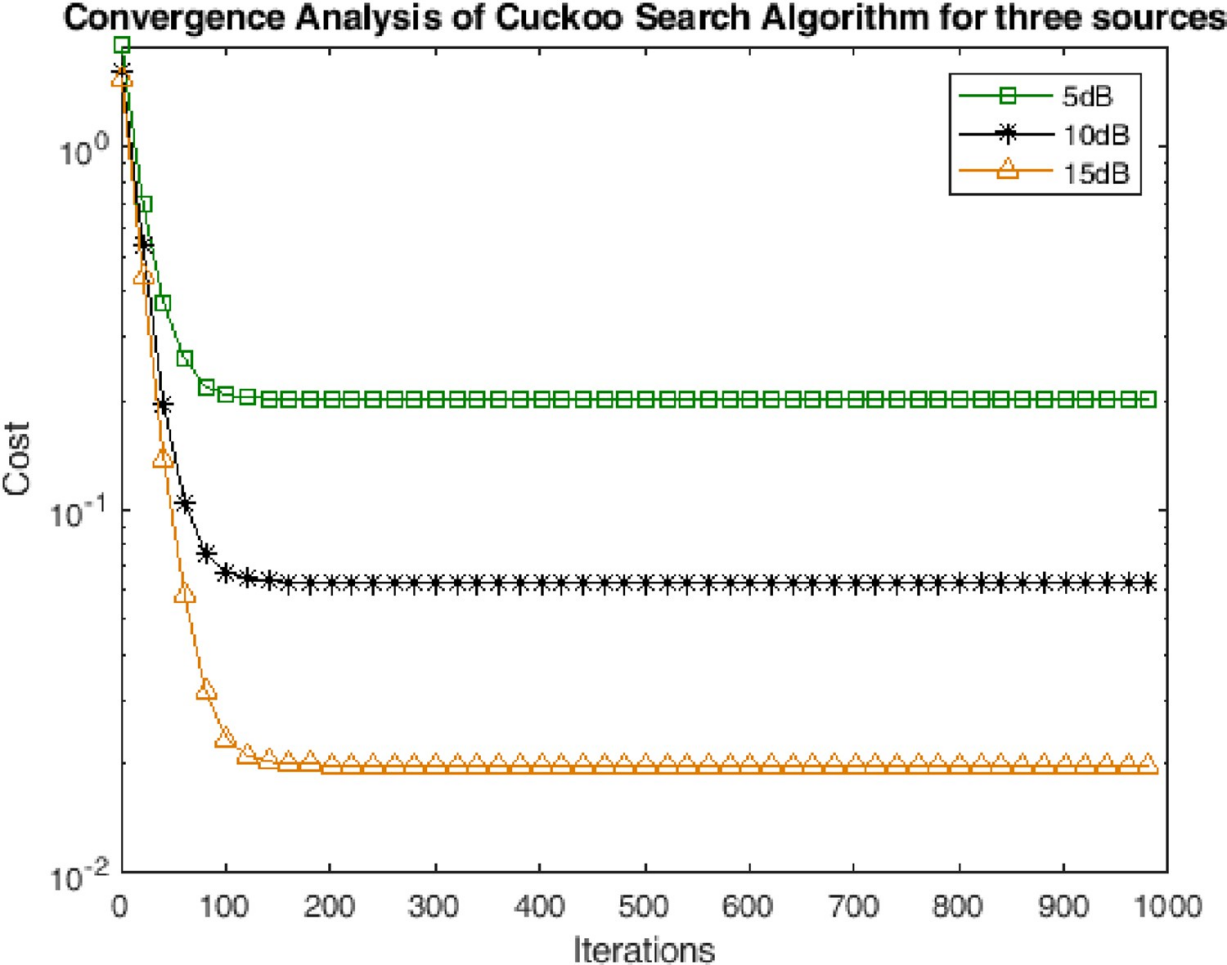
Convergence analysis for three sources.

**Fig 5:**
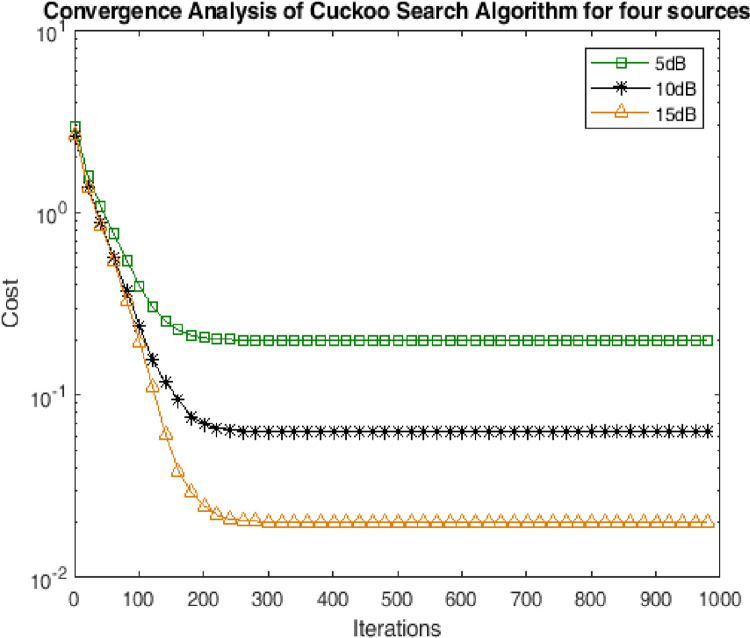
Convergence analysis for four sources.

In this subsection the performance has been assessed in terms of convergence of the CSA towards solution. The performance has been measured for multiple scenarios of noise. It can be seen from the Figs 3–5 shows that the CSA algorithm converged in 50 iterations for two sources. Moreover, the CSA converged at 100 and 200 iterations for three and four sources respectively. The very earlier convergence of the algorithm reveals the strength of the algorithm for the optimization of highly non-linear cost function of DOA of underwater multi-targets.

### 4.3. Robustness against noise

This subsection describes the trend of RMSE against the linearly varying level of SNR. The convergence analysis has been analyzed in various cases of targets.

**Fig 6:**
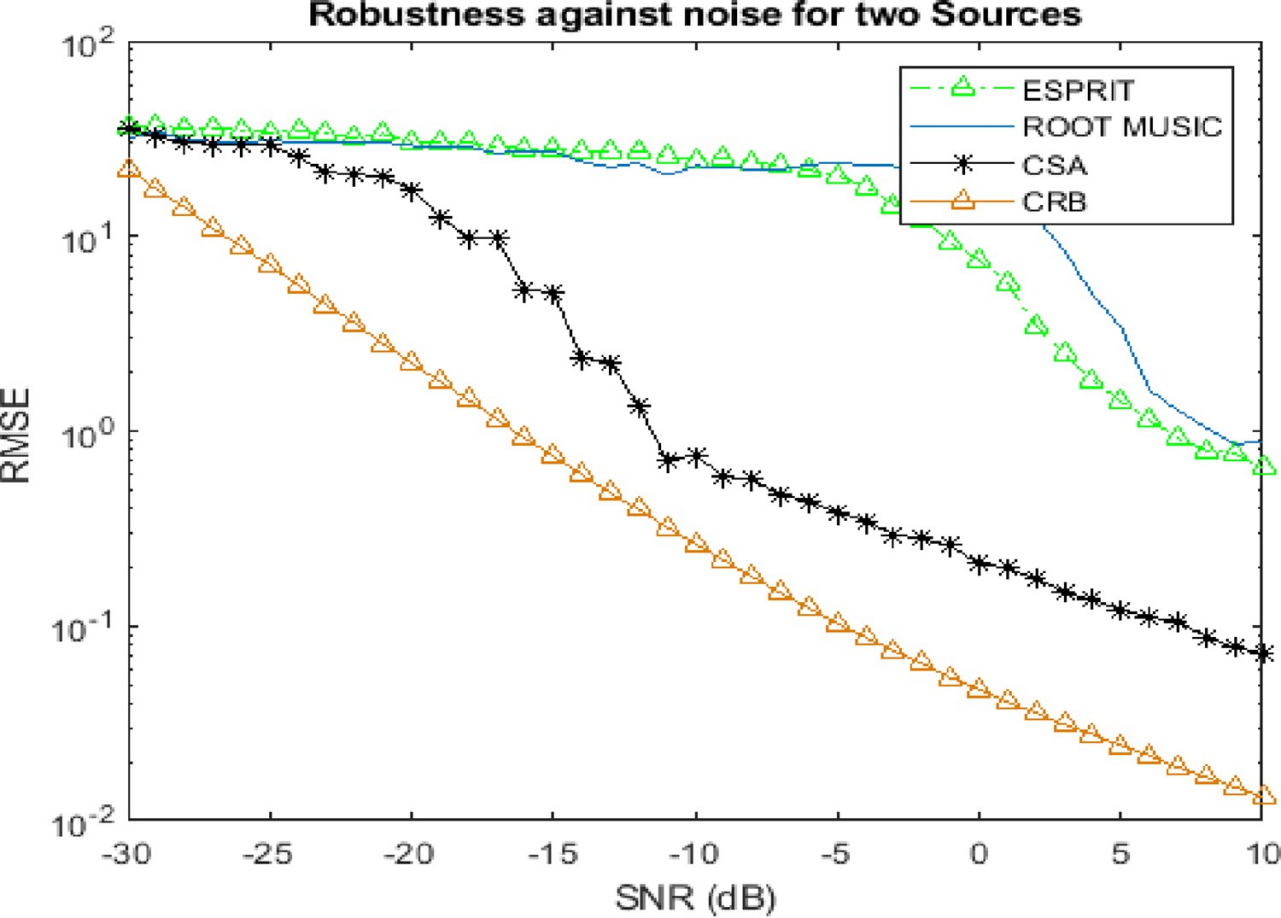
Robustness against noise for two sources.

**Fig 7:**
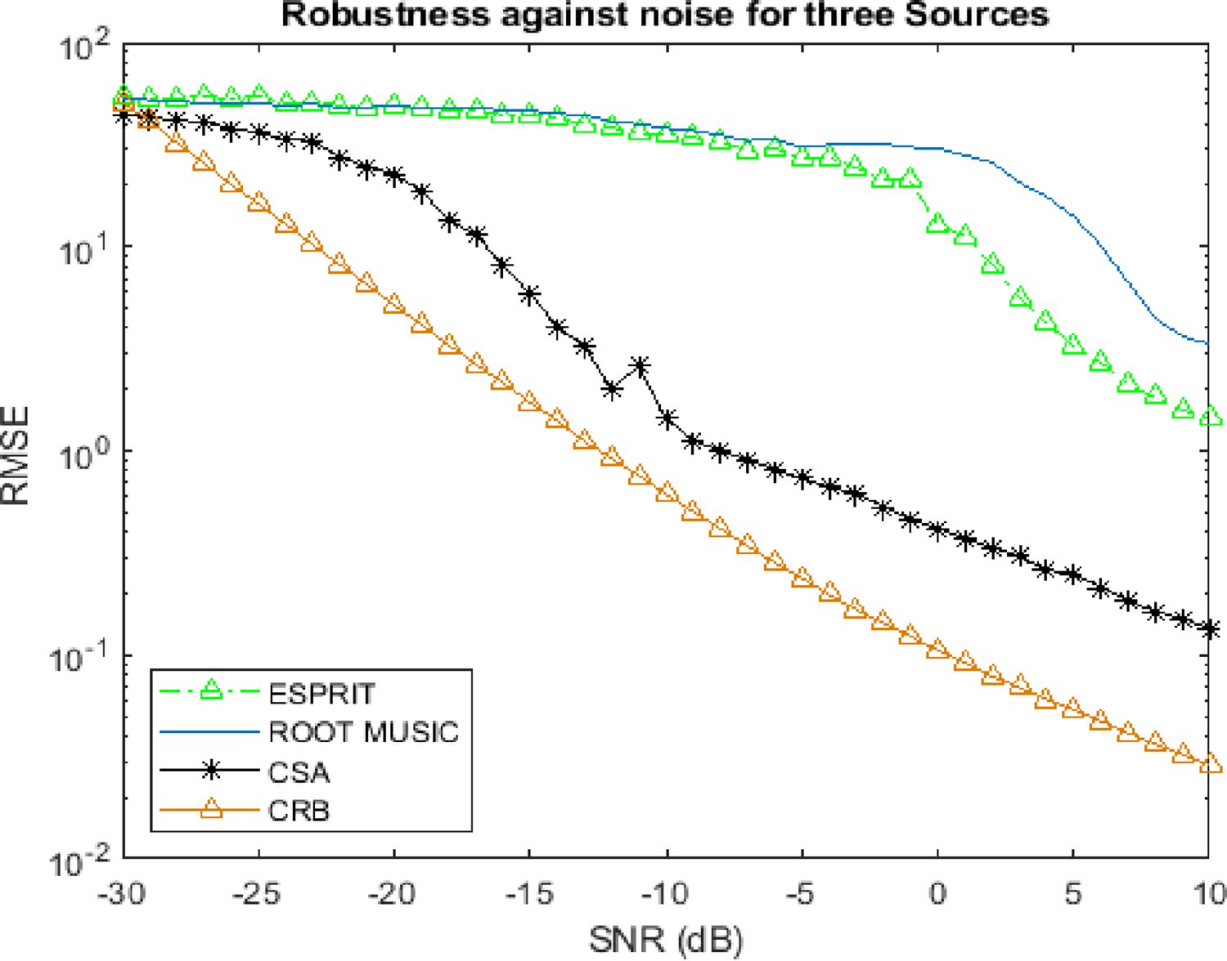
Robustness against noise for three sources.

Figs 6 and 7 reveals that the convergence trend of RMSE of CSA algorithm against low SNR is exclusively far better than Root MUSIC and ESPRIT algorithms which have been also validated by CRB by taking 300 Monte Carlo trials.

### 4.4. Frequency distribution of RMSE

**Fig 8:**
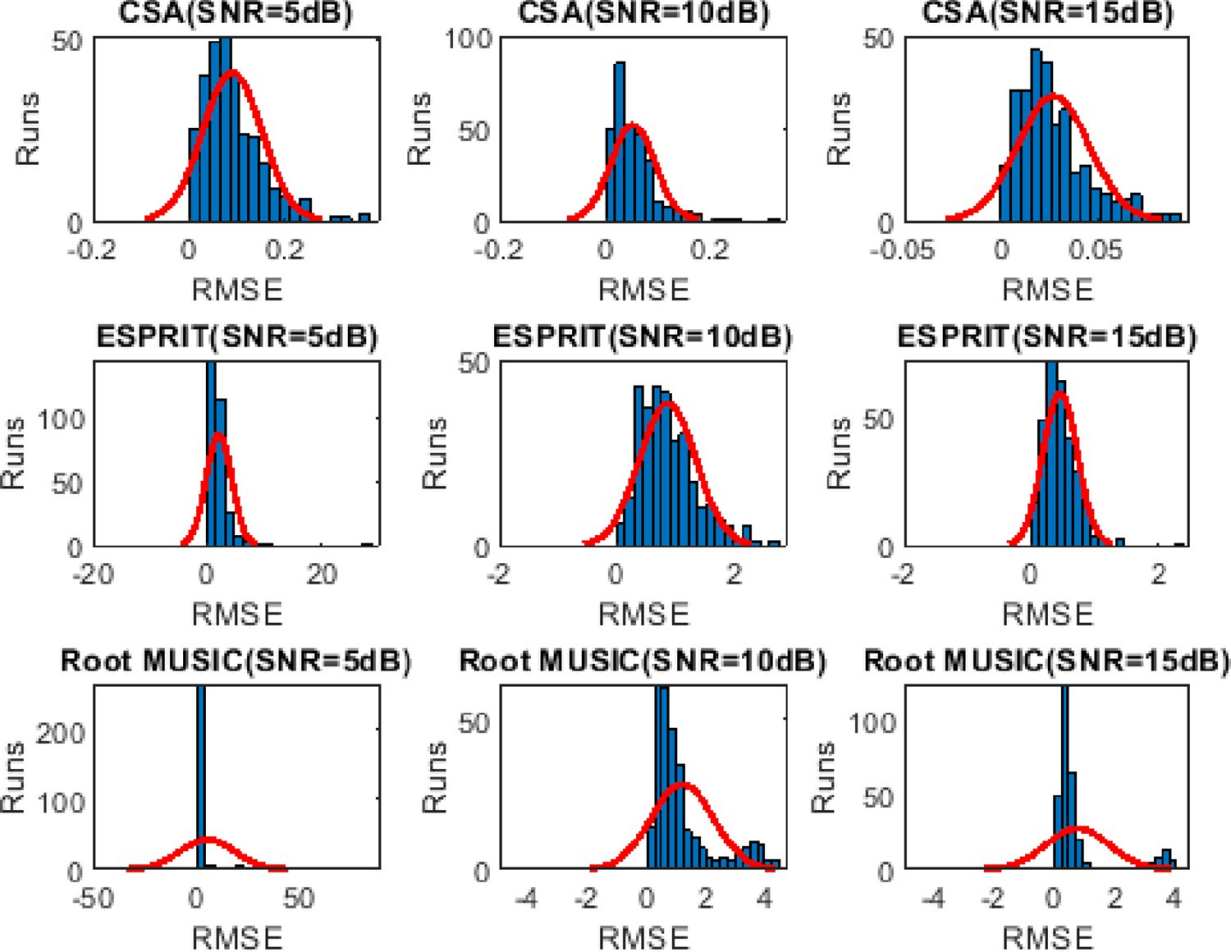
Histogram analysis of two sources for 300 Monte carlo runs.

**Fig 9:**
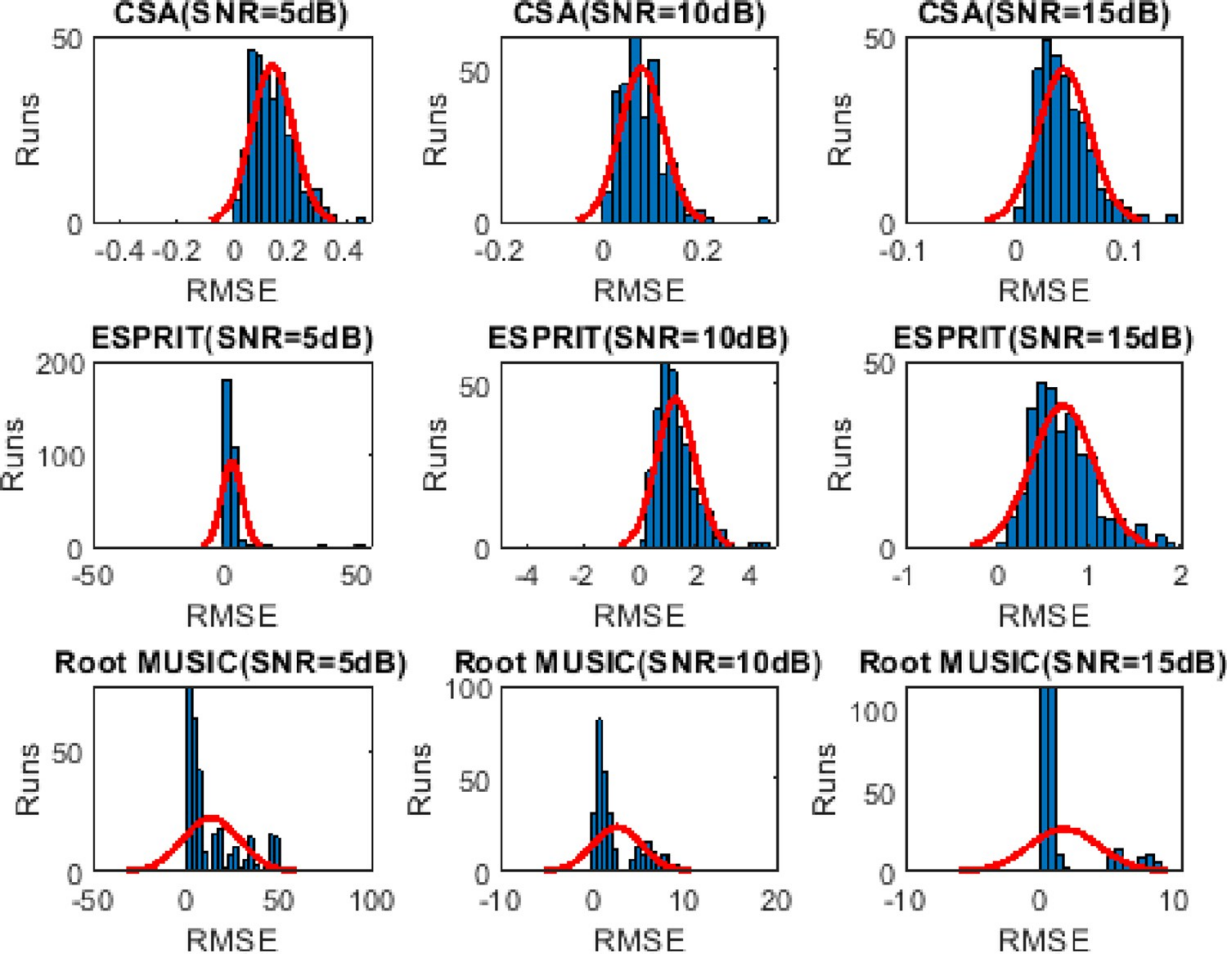
Histogram analysis of three sources for 300 Monte carlo runs.

**Fig 10:**
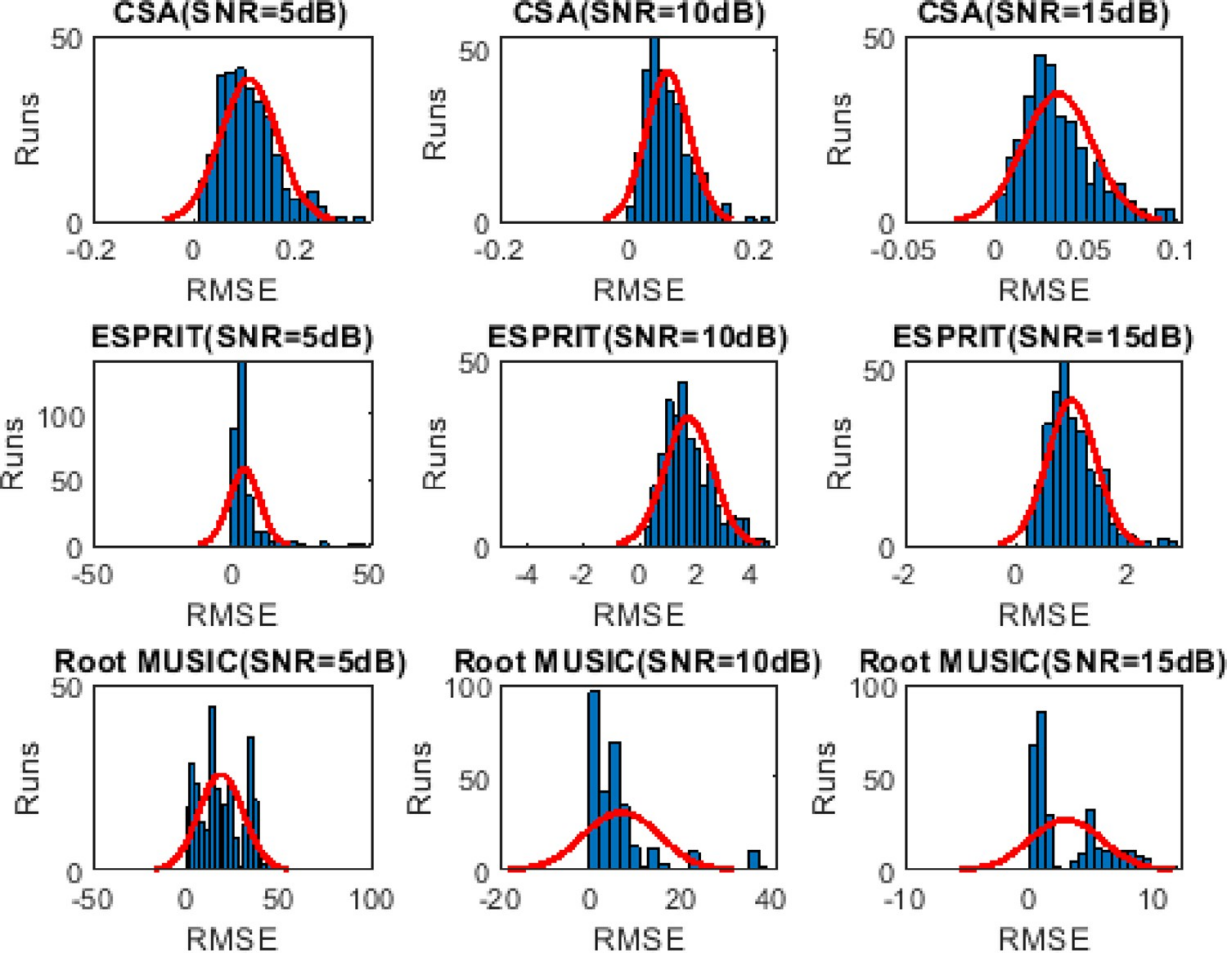
Histogram analysis of four sources for 300 Monte Carlo runs.

Histogram analysis describes the frequent successful happenings (trials with minimum RMSE value) over the Monte Carlo trials. Wider shape depicts lesser frequency distribution. It can be seen from Figs 8–10 that the frequency of the minimum valued RMSE is higher for CSA as compared to the ESPRIT and Root MUSIC algorithm.

### 4.5. Empirical CDF of RMSE

**Fig 11:**
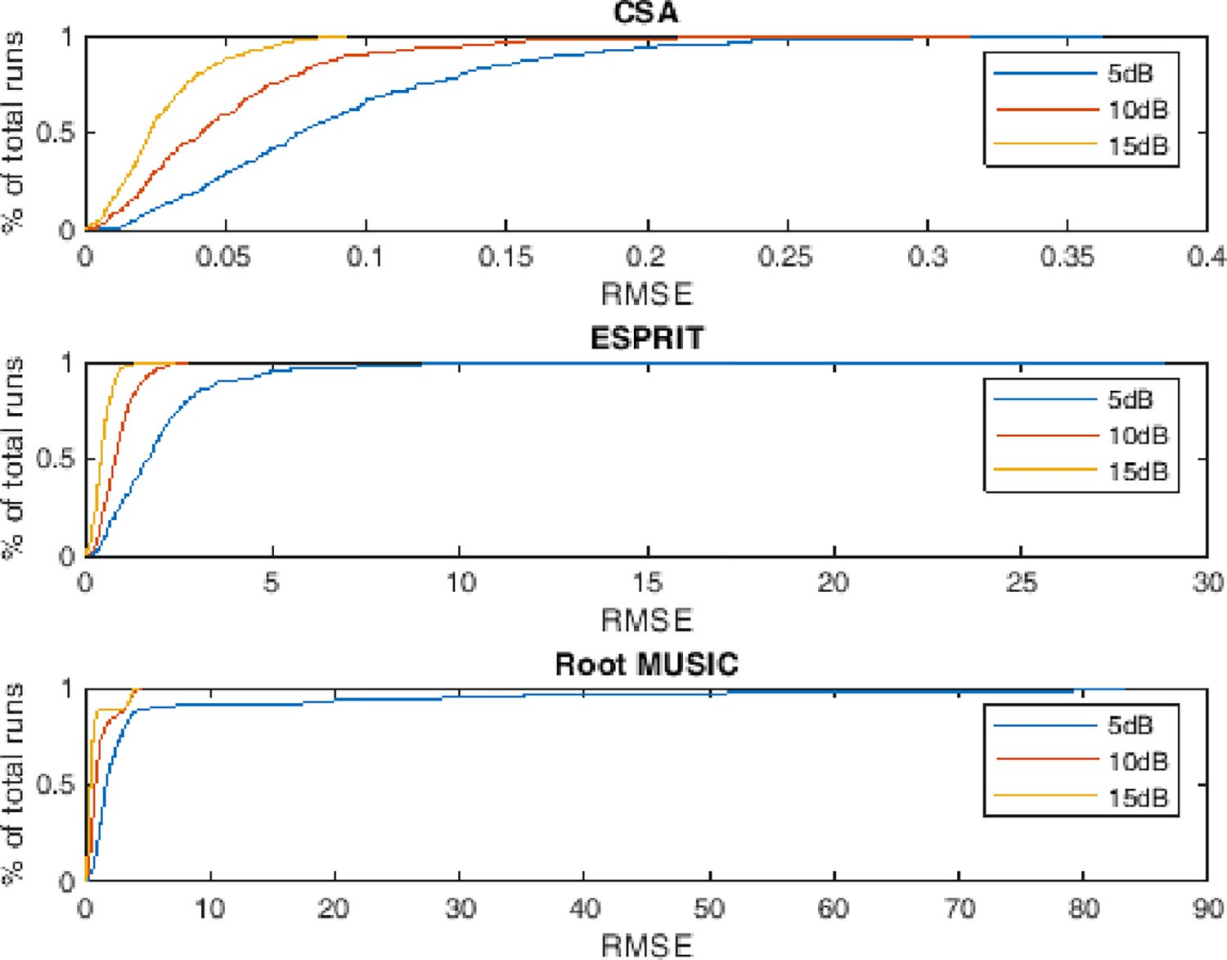
Empirical cumulative distribution function of RMSE for two sources.

**Fig 12:**
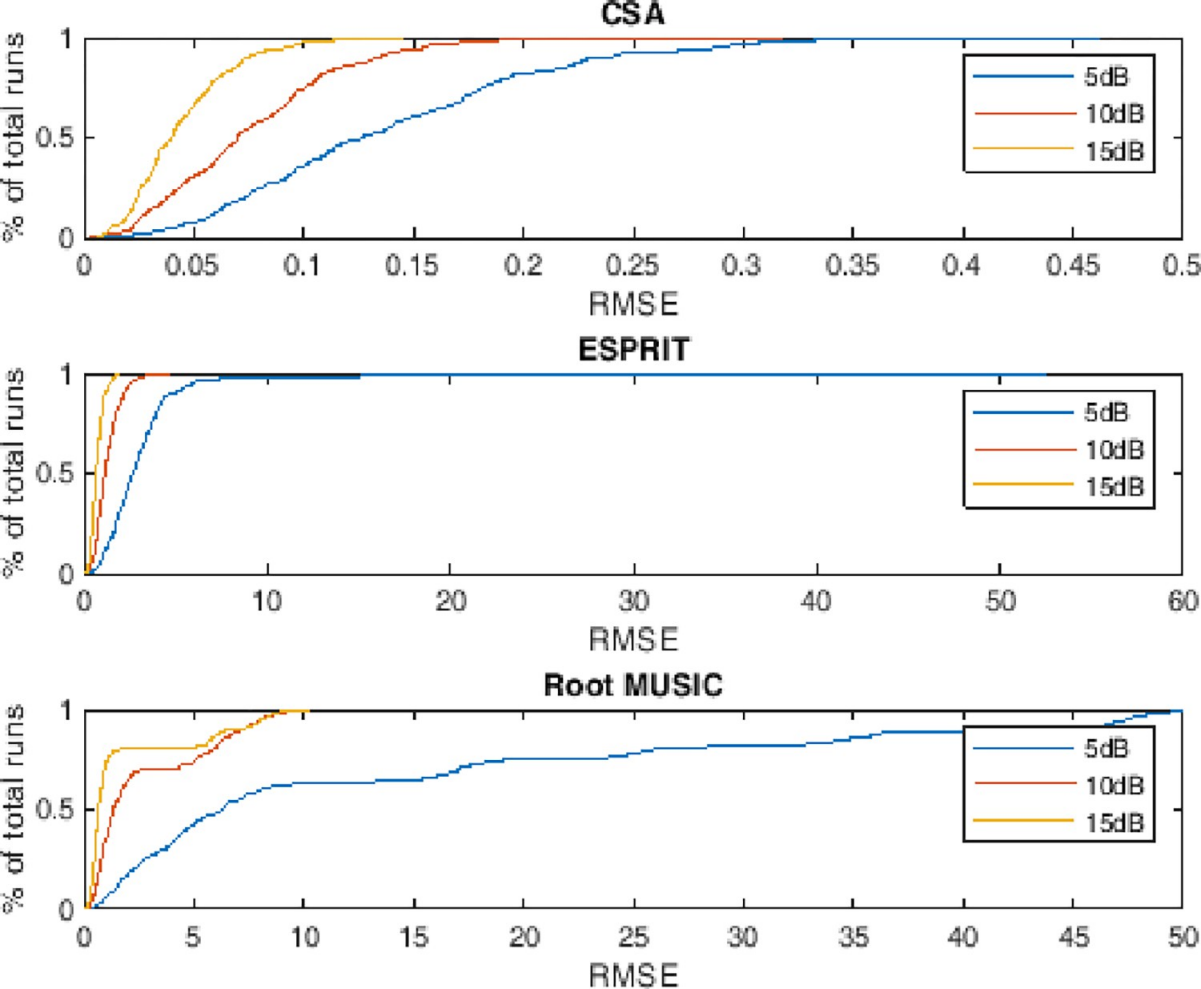
Empirical cumulative distribution function of RMSE for three sources.

**Fig 13:**
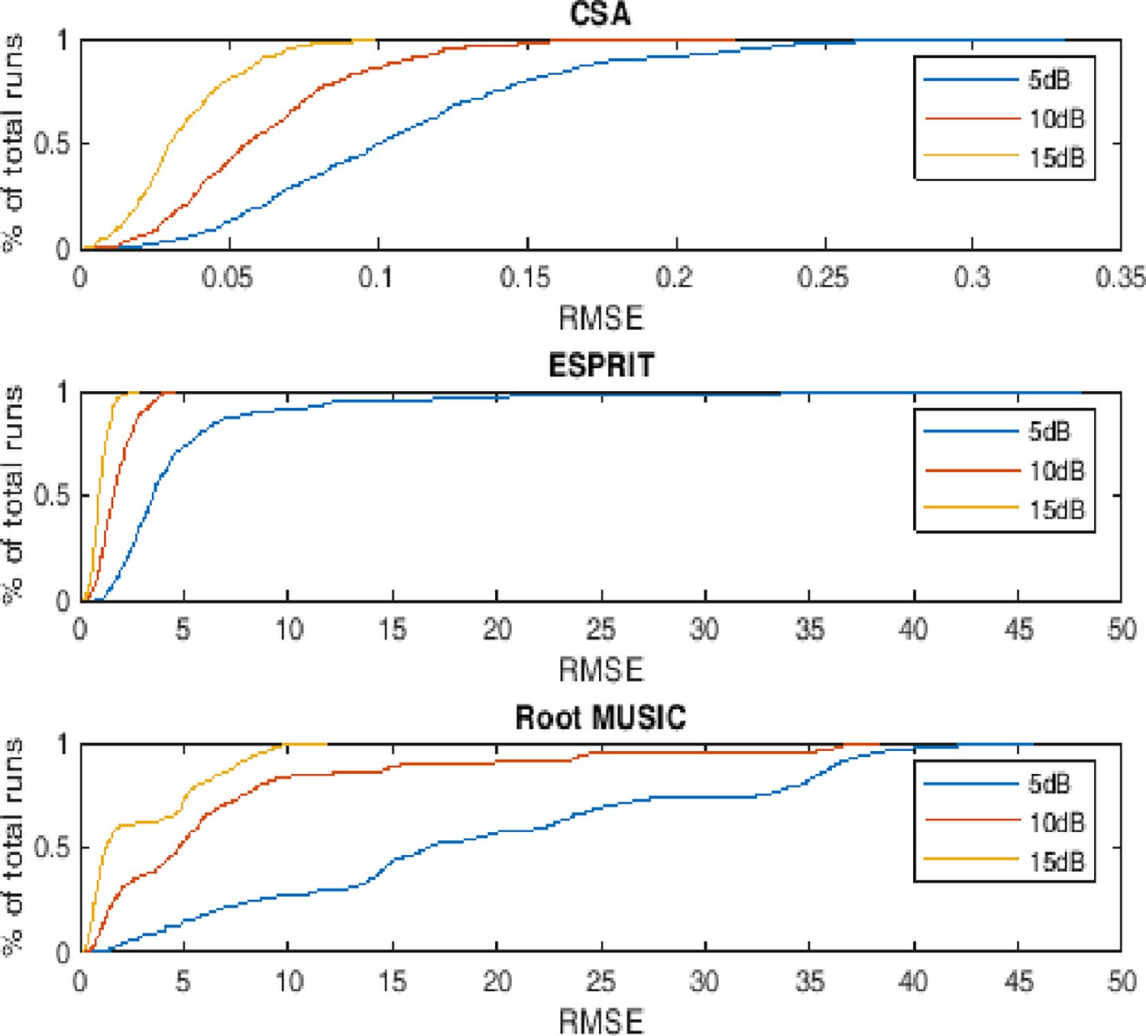
Empirical cumulative distribution function of RMSE for four sources.

This subsection describes the probability distribution of RMSE against the monte carlo run for two, three and four targets having different level of noise. The performance metric also depicts the dynamics of RMSE. It can be evaluated that more than 90 percent of the runs are with minimum valued RMSE against the state of the art counterparts i.e. ESPRIT and Root MUSIC as shown in Figs 11–13.

## 4.6. Variability analysis of RMSE

**Fig 14:**
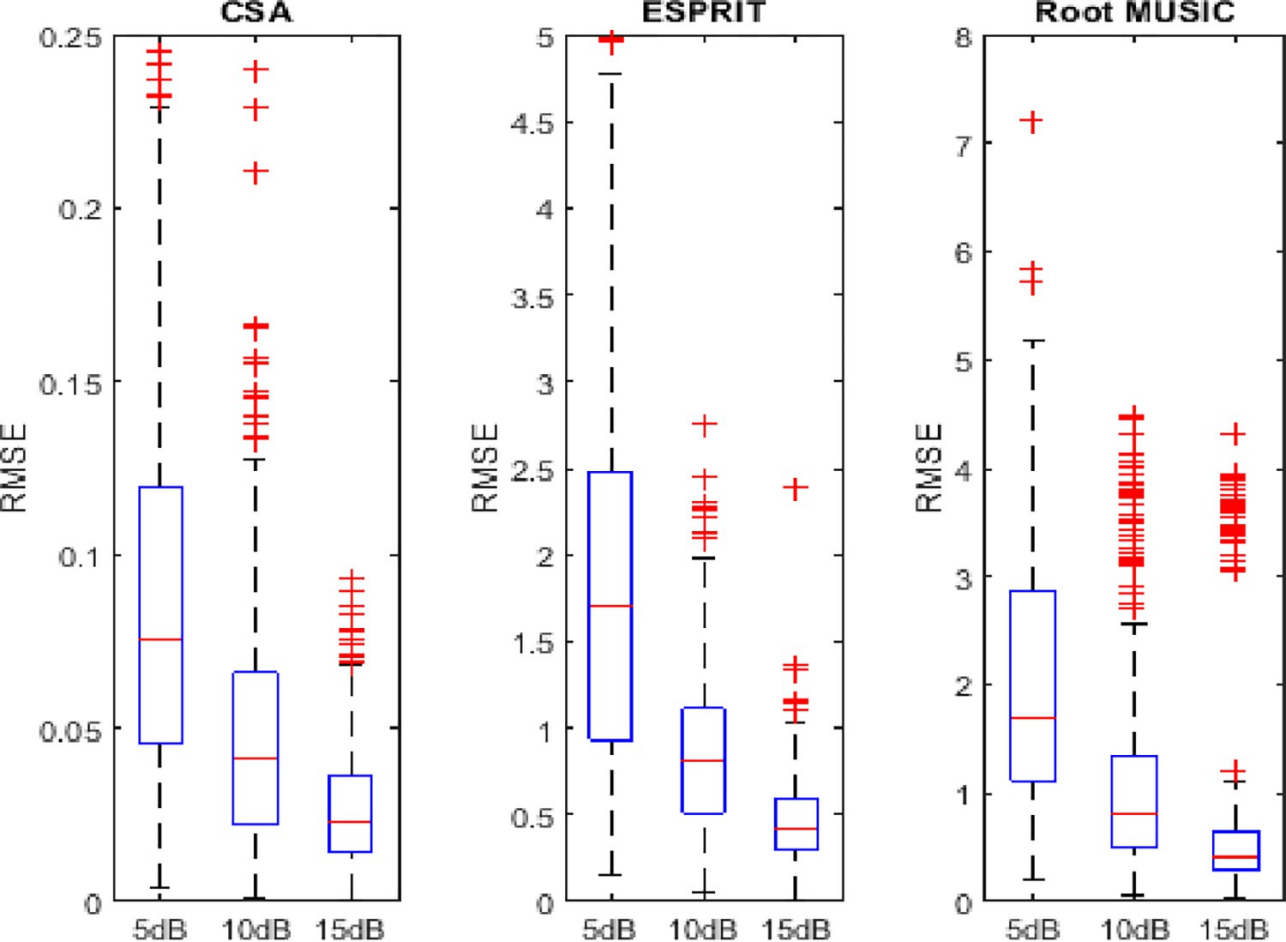
Variability analysis of RMSE for two sources.

**Fig 15:**
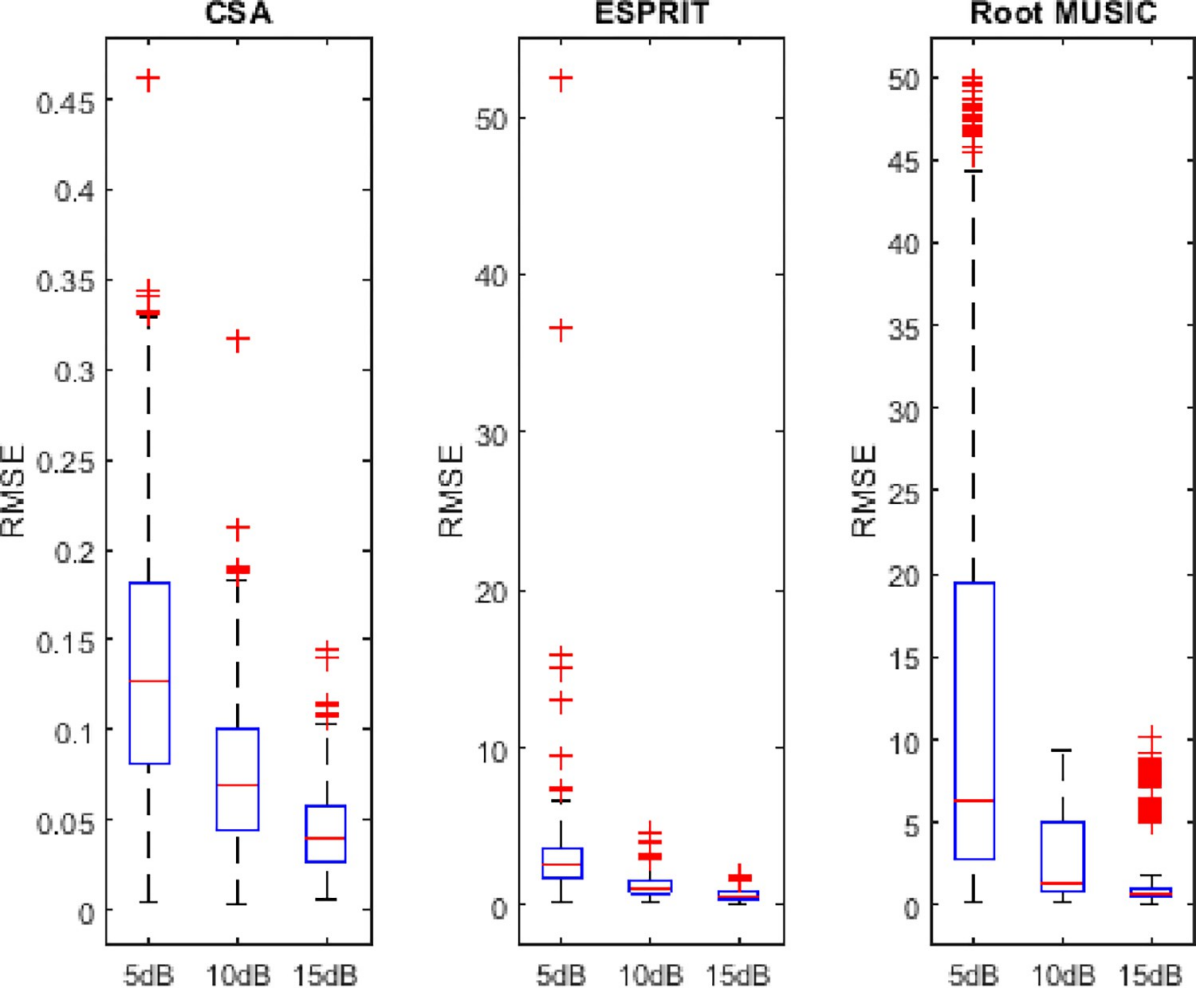
Variability analysis of RMSE for three sources.

**Fig 16:**
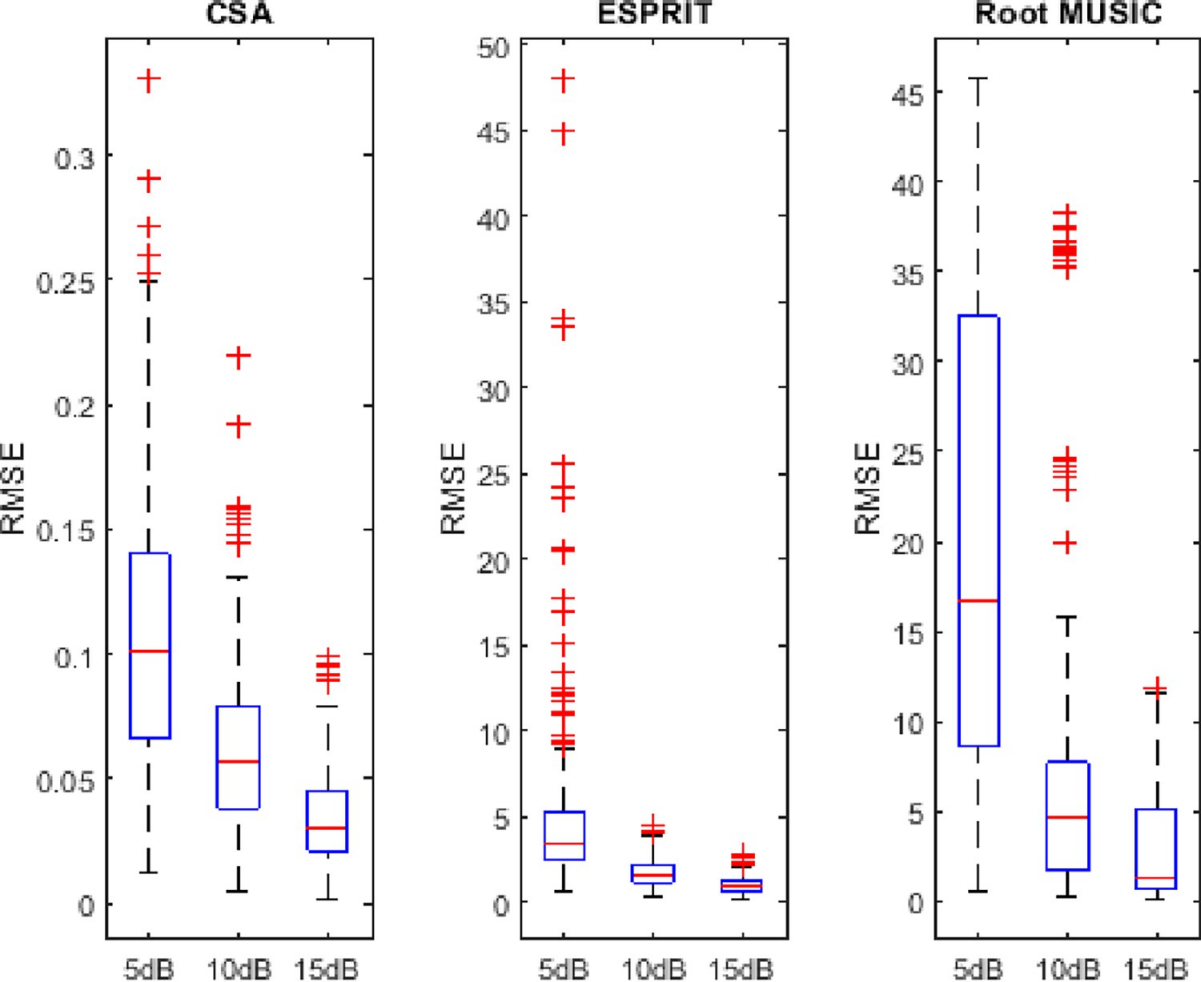
Variability analysis of RMSE for four sources.

In this subsection, the sorted RMSE observations are distributed in five quartiles (minimum to first 25 percent observations and from 25 to 75 percent and from 75 percent to above). The first and the last observation is the minimum and maximum value of the RMSE, respectively. This distribution gives an insight into the performance of algorithms. The symbol + denotes the outliers (abnormalities) in the observations. It can be found from Figs 14–16 that the minimum spread out depicts the consistency of algorithms. The dispersion rate of the CSA is less as compared to state of the art algorithms i.e. RMSE is about 0.045 to 0.11 for 5dB of SNR. Moreover, asymmetry is depicted by the outliers in the figure. The RMSE of the CSA is symmetric as compared to ESPRIT and Root MUSIC.

### 4.7. Resolution ability for closely spaced targets

**Fig 17:**
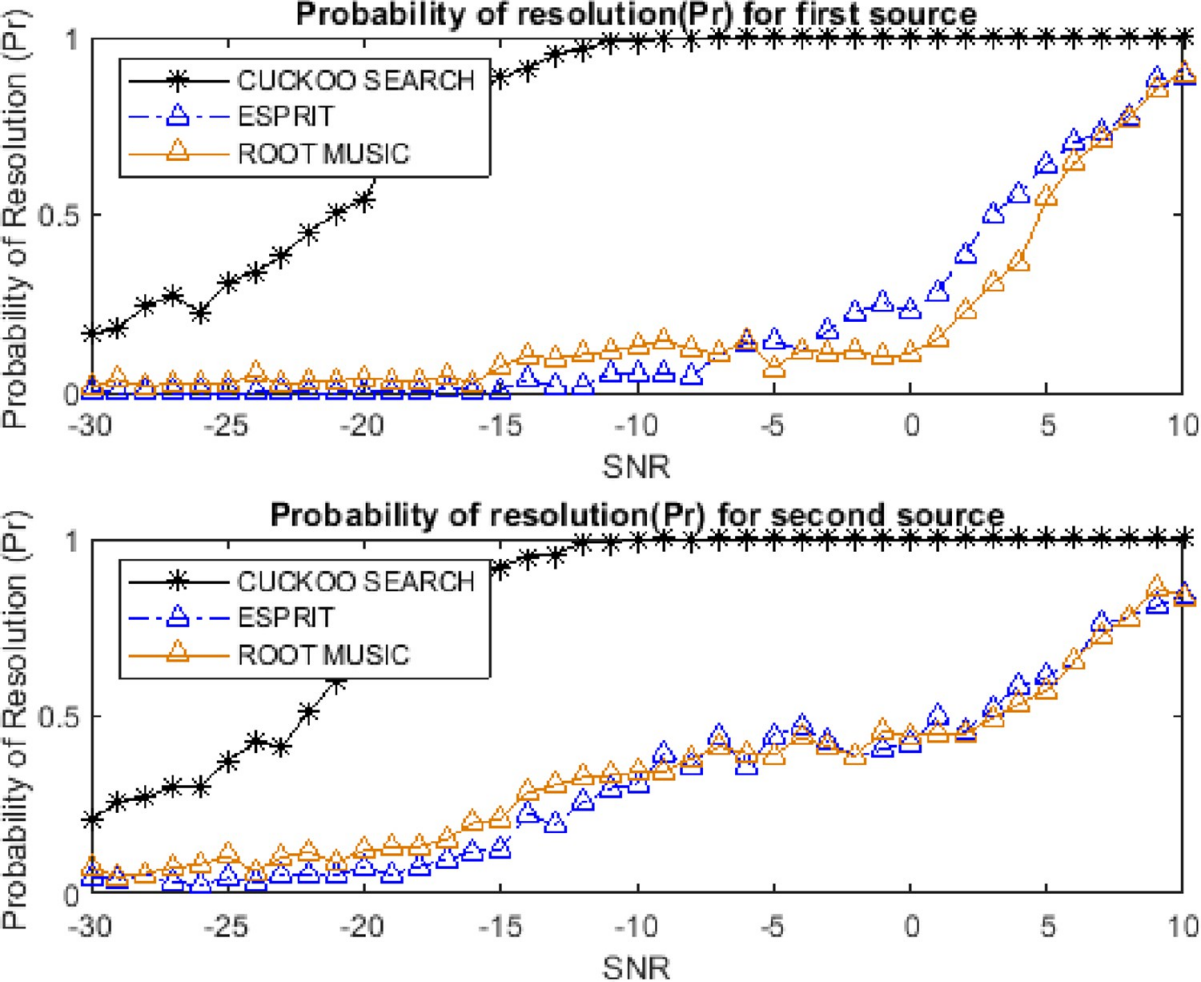
Probability of resolution for two sources for 4 degrees separation.

**Fig 18:**
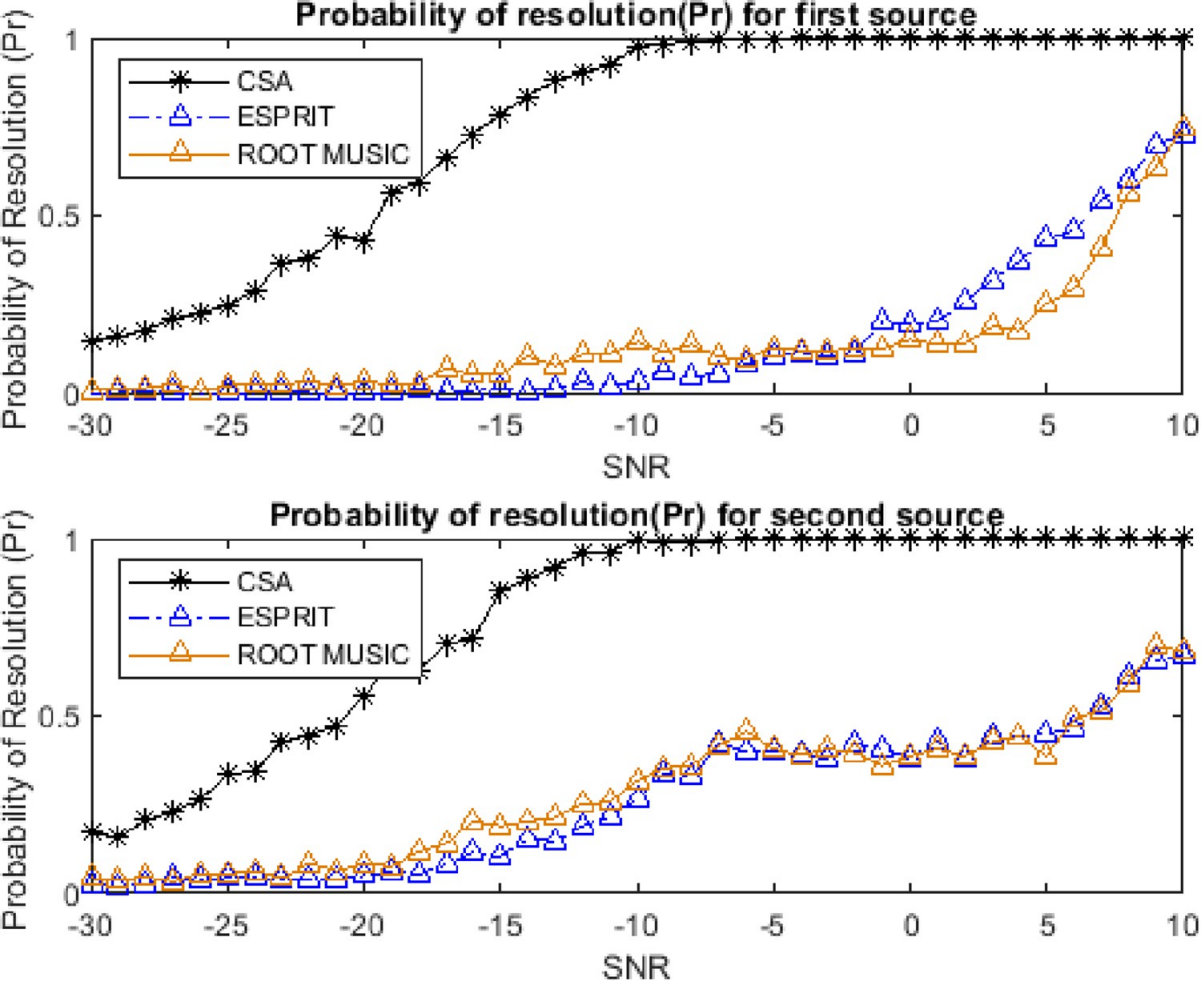
Probability of resolution for two sources for 3 degrees separation.

The resolution ability is one of the important performance parameters to analyze the closely spaced targets. The probability of resolution can be defined as:

$$P_{r}=Prob[|\theta_{e}-\theta_{a}|\leq \frac{△\theta }{2}]$$

where △θ = |θ_1_−θ_2_|. The resolution ability of both closely spaced sources is shown in Figs 17 and 18. The performance has been evaluated for two different separations between two sources. The number of successful runs (the runs having RMSE less than the threshold) is higher for the CSA algorithm as compared to ESPRIT and Root MUSIC algorithms.

## 5. Potential use and limitations

Acoustic plane waves mostly suffer in the low SNR in underwater environment due to the far-field regime. Consequently, the estimation accuracy becomes an indespensible challenge for DOA estimation and target tracking in underwater multi-sources. In the results and discussion section, it can be seen that the state of the art counterparts i.e. MUSIC, ESPRIT and RMUSIC deteriotes in terms of estimation accuracy with high RMSE. Statistical analysis of the RMSE in Monte Carlo trials i.e. ECDF of RMSE, variability analysis of RMSE, frequency distribution of RMSE and the probability of resolution witnesses the strength of CSA in this challenging environment. Moreover, the computation complexity will become a trade off for the large number of snapshots exclusively for swarming intelligent algorithms.

## 6. Conclusions

Estimation accuracy and resolution ability are the main challenges in DOA estimation for closely spaced targets and CSA outperforms its counterpart algorithms (ESPRIT and Root MUSIC) in these performance measures. To be more specific, the state of the art counterparts i.e. MUSIC, ESPRIT and RMUSIC deteriotes in terms of estimation accuracy with high RMSE. Statistical analysis of the RMSE in Monte Carlo trials i.e. ECDF of RMSE, variability analysis of RMSE, frequency distribution of RMSE and the probability of resolution witnesses the strength of CSA in this challenging environment of low SNR and less number of snapshots. In the future, estimation of 2D-DOA using a uniform circular array is planned using the CSA algorithm and other modern computing paradigms [43–47].

## Supporting information

S1 Data (ZIP)
